# Inverse Association between Organic Food Purchase and Diabetes Mellitus in US Adults

**DOI:** 10.3390/nu10121877

**Published:** 2018-12-03

**Authors:** Yangbo Sun, Buyun Liu, Yang Du, Linda G. Snetselaar, Qi Sun, Frank B. Hu, Wei Bao

**Affiliations:** 1 Department of Epidemiology, College of Public Health, University of Iowa, Iowa City, IA 52242, USA; yangbo-sun@uiowa.edu (Y.S.); buyun-liu@uiowa.edu (B.L.); yang-du-1@uiowa.edu (Y.D.); linda-snetselaar@uiowa.edu (L.G.S.); 2 Department of Nutrition, Harvard T.H. Chan School of Public Health, Boston, MA 02115, USA; qisun@hsph.harvard.edu (Q.S.); fhu@hsph.harvard.edu (F.B.H.); 3 Channing Division of Network Medicine, Department of Medicine, Brigham and Women’s Hospital and Harvard Medical School, Boston, MA 02115, USA; 4 Department of Epidemiology, Harvard T.H. Chan School of Public Health, Boston, MA 02115, USA; 5 Obesity Research and Education Initiative, University of Iowa, Iowa City, IA 52242, USA; 6 Fraternal Order of Eagles Diabetes Research Center, University of Iowa, Iowa City, IA 52242, USA

**Keywords:** organic food purchase, diabetes, NHANES, cross-sectional, population-based

## Abstract

Background: The organic food market has grown rapidly worldwide in the past 15 years. However, evidence concerning the health effects of organic foods is scarce. We evaluated the cross-sectional association of organic food purchase, as a proxy of organic food consumption, with diabetes in a nationally representative population. Methods: We included 8199 participants aged ≥20 years from the National Health and Nutrition Examination Survey 2007–2008 and 2009–2010. Organic food purchase and frequency were ascertained by questionnaires. Diabetes was defined as a self-reported physician diagnosis or a hemoglobin A1c level ≥6.5% or both. We used logistic regression with sample weights to estimate the odds ratios (ORs) and 95% confidence intervals (CIs). Results: Individuals who reported purchasing organic foods were less likely to have diabetes compared to those who did not report organic food purchase. After adjustment for age, gender, race/ethnicity, family history of diabetes, socioeconomic status, and dietary and lifestyle factors, the OR of diabetes associated with organic food purchase was 0.80 (95% CI 0.68–0.93). The association remained significant after additional adjustment for BMI with OR of 0.80 (0.69–0.94). Conclusions: In a nationally representative population, frequent organic food purchase was inversely associated with diabetes prevalence in adults in the United States.

## 1. Introduction

Diabetes is one of the major causes of morbidity and mortality in the United States, and has extensive financial burden [1]. By 2040, it will affect as many as 642 million people [2]. In recent years, accumulating evidence has highlighted the importance of modifiable risk factors, including diet and environmental factors, in the prevention of diabetes [3]. Reduced exposure to environmental chemical contaminants [3] and increased consumption of favorable nutrients [4], such as polyunsaturated fatty acids, polyphenolics, and antioxidants, have been demonstrated to be associated with a lower risk of diabetes in humans. Organic foods seem to be a good candidate for preventing diabetes because they possess both of the aforementioned benefits.

Organic foods are produced by methods that comply with organic standards, generally restricting the use of agrochemicals (e.g., pesticides, synthetic soluble fertilizers, antibiotics, and sex steroids or growth hormones, etc.) [5]. For example, in the U.S., organically grown foods consistently have about one-third as much pesticide residues as conventionally grown foods [6]. Moreover, organic foods contain a higher content of beneficial nutritional compositions, including polyphenolics, antioxidants, and polyunsaturated fatty acids, etc. [7,8]. Health benefits are the main motivation for the consumption of organic foods [5]. However, studies concerning the health effects of organic foods in humans are scarce [5,9,10]. Furthermore, there is an argument that people who purchase organic foods maintain a healthier lifestyle including a healthier diet, etc., potentially reducing the risk of several major diseases [11], and whether consumption of organic foods has an additional positive influence on health is still under scientific debate. The U.S market for organic foods has grown from $3.5 billion in 1996 to $18 billion in 2007 and continued to grow to $35 billion in 2014 [12,13]. With the rapidly growing organic food market in the U.S., it is important to understand whether organic food consumption will reduce the risk of major chronic diseases such as diabetes, and furthermore whether the effects are independent of socioeconomic status and other risk factors, such as race/ethnicity, family history of diabetes, smoking, physical activity, and dietary quality.

To address these questions, we used data from a nationally representative population to examine the association of organic food purchases, as a proxy of organic food consumption, and the frequency of purchasing total and individual organic foods with diabetes prevalence in U.S. adults.

## 2. Materials and Methods

### 2.1. Study Population

The study population consisted of participants from the 2007–2008 and 2009–2010 cycles of the National Health and Nutrition Examination Survey (NHANES) as the purchase of organic foods was only evaluated in these two cycles. Briefly, the NHANES is a large-scale, ongoing, nationally representative health survey of the non-institutionalized U.S. population. It is conducted by the National Center for Health Statistics (NCHS) of the Centers for Disease Control and Prevention (CDC). NHANES survey data are released every two years; each cycle consists of approximately 10,000 participants [14]. The data comprises population-based, cross-sectional surveys about diet, nutritional status, general health, disease history, and health behaviors [14]. Most questionnaire data are collected during in-house interviews, while health examinations and dietary interviews are performed in specially-designed and equipped mobile examination centers that travel to locations throughout the country. The surveys use multi-stage, probability clusters to develop a population sample that is nationally representative of the U.S. based on age, sex, and race/ethnicity. NHANES data along with documents on the survey methods and other information are publicly available on the NHANES website [15]. The NHANES has been approved by the National Center for Health Statistics Ethics Review Board. All subjects gave written informed consent. Due to the use of de-identified data, the University of Iowa institutional review board determined that the current study was exempt (IRB # 201706805).

In 2007–2008 and 2009–2010, 12,028 non-pregnant adults aged ≥20 years were initially included. After applying the exclusion criteria, we finally included 8199 participants in this analysis (Flowchart shown in the Figure S1).

### 2.2. Exposure Measurement

Only participants who completed a dietary interview in the mobile examination center were eligible to participate in the Flexible Consumer Behavior Survey follow-up interview, in which the questionnaire item about organic food purchase was asked.

The primary exposure of our study was purchase of organic foods. For both the 2007–2008 and 2009–2010 cycles, during the interview, participants were asked: “In the past 30 days, did you buy any food that had the word ’organic’ on the package?”

The secondary exposure was the frequency of organic food purchase. For the 2007–2008 cycle, participants were further asked, “How often do you buy organic food? Would you say always, most of the time, sometimes, or rarely?” For the 2009–2010 cycle, the frequency of purchase of individual organic food items (fruits, vegetables, milk and other dairy products, eggs, poultry, and meats) was asked separately. For example, for fruits, the question was, “In the past 30 days, when you bought fruits, how often did you buy organic fruits? Would you say always, most of the time, sometimes, rarely, or never?” Participants who responded with “always” and “most of the time” were combined as “most of the time/always” due to the relatively small sample size in these two categories.

### 2.3. Outcome Measurement

Participants were classified as having diabetes if they reported having been previously diagnosed with diabetes (for women other than during pregnancy) by a doctor or health care professional. Participants who were not previously diagnosed with diabetes but had hemoglobin A1C ≥ 6.5% were also classified as having diabetes [16,17,18]. Hemoglobin A1c levels were measured using blood samples collected during the health examination according to a standardized protocol. The inter-assay coefficient of variation was 0.8% to 3.7% for glucose levels and 0.7% to 3.1% for HbA1c levels [1,19].

### 2.4. Covariate Assessment

Information on age, gender, race/ethnicity, education, annual household income, family history of diabetes, smoking status, and physical activity was obtained during the interview [20]. Race/ethnicity was categorized as non-Hispanic white, non-Hispanic black, Hispanic (Mexican and non-Mexican Hispanic), and other race/ethnicity. Education was grouped as less than high school, high school, and college or higher. Family income-to-poverty ratios were categorized as ≤1.30, 1.31–3.50, and >3.50 [21]. Individuals who smoked less than 100 cigarettes in their lifetime were defined as never smokers; those who had smoked more than 100 cigarettes but did not smoke at the time of the survey were considered former smokers; those who had smoked more than 100 cigarettes and smoked cigarettes at the time of the survey were current smokers [22]. Dietary intake was assessed through two 24-h dietary recalls. Total energy intake and alcohol intake were calculated using the food composition database. Overall diet quality was indicated by the Healthy Eating Index (HEI) 2010, which has been previously associated with a lower risk of diabetes [23]. It is scored on the basis of the intake levels of 12 dietary components including total fruit, whole fruit, total vegetables, greens and beans, whole grains, dairy, total protein foods, seafood and plant proteins, fatty acids, refined grains, sodium, and empty calories (i.e., energy from solid fats, alcohol, and added sugars) [24]. Alcohol intake was categorized as non-drinking (0 g/day), moderate drinking (0.1–27.9 g/day for men and 0.1–13.9 g/day for women), and heavy drinking (≥28 g/day for men and ≥14 g/day for women). Physical activity was assessed using the Global Physical Activity Questionnaire. We classified physical activity into three groups (<600, ≥600–1199, and ≥1200 metabolic equivalent (MET)-min/week). Measurements of height and weight were performed following a standardized protocol, and body mass index (BMI) was computed as weight in kilograms divided by the square of height in meters.

### 2.5. Statistical Analysis

All statistical analyses accounted for the complex, multistage, stratified, cluster-sampling design of NHANES by using sample weights, strata, and primary sampling units embedded in the NHANES data. Comparisons of characteristics according to organic food purchase in the past 30 days were performed using the t-test for continuous variables and the chi-square test for categorical variables.

We used multivariable logistic regression to estimate odds ratios (ORs) and 95% confidence intervals (CIs) of diabetes in relation to organic food purchase and the frequency of organic food purchase. In multivariable models, we adjusted for age, gender, race/ethnicity, education, family income-to-poverty ratio, family history of diabetes, smoking status, alcohol intake, physical activity, total energy intake, HEI-2010 score, and BMI. For individual organic food, we also adjusted for the total consumption of the corresponding food group; for example, for organic fruits, we also adjusted for the consumption of total fruits in the model.

To evaluate the potential effect modification, we performed stratified analyses according to age (20–44, 45–64, and ≥65 years old), gender (male vs. female), race/ethnicity (white and non-white [non-Hispanic black, Hispanic, and others]), education (less than high school, high school, and college or above), ratio of family income to poverty (≤1.30, 1.31–3.50, and >3.50) [21], physical activity levels (<600, 600–1199, and ≥1200 MET-min/week), and diet quality (lower diet quality (below mean HEI-2010 score) vs. higher diet quality (above mean HEI-2010 score)). We conducted interaction tests via multiplicative interaction terms in the multivariable models. To reduce the concern of reverse causation (i.e., participants started to use organic foods after being diagnosed with diabetes), we did a sensitivity analysis restricted to participants with underlying diabetes who were not previously diagnosed but discovered by glucose and HbA1c tests during the NHANES examination. Although NHANES does not explicitly collect information on diabetes subtypes, we did a sensitivity analysis excluding those who possibly had type 1 diabetes, if they started insulin within one year of diabetes diagnosis, were currently using insulin, and were diagnosed with diabetes under age 30 [25]. All analyses were performed using survey procedures in SAS 9.4 (SAS Institute, Cary, NC, USA).

## 3. Results

We included 8199 participants in this study with an average age of 49.7 years (standard error [SE] = 0.36). We identified 343 cases of diabetes among 2899 participants who purchased organic food and 875 cases of diabetes among 5300 participants who did not purchase any organic foods. Participants who purchased organic foods in the past 30 days were more likely to be younger, female, white, with higher education and higher family income, without a family history of diabetes, non-smoker, with more alcohol intake and higher physical activity levels, with less total energy intake and higher overall diet quality, and with lower BMI (Table 1).

Participants who purchased organic foods were less likely to have diabetes compared to those who did not. After adjustment for age, gender, race/ethnicity, socioeconomic status, family history of diabetes, and dietary and lifestyle factors, the multivariable-adjusted OR of diabetes associated with organic food purchase was 0.80 (95% CI, 0.68–0.93) (Table 2). The association remained significant after additional adjustment for BMI, with a multivariable-adjusted OR of 0.80 (95% CI, 0.69–0.94). The multivariable-adjusted ORs (95% CIs) were 0.96 (0.71–1.30), 0.84 (0.59–1.21), and 0.72 (0.35–1.45) for participants who purchased organic foods rarely, sometimes, and most of the time/always, respectively, compared with participants who never purchased organic foods (Table 3). The associations remained similar after an additional adjustment for BMI.

The association of the frequency of purchase of organic foods with risk of diabetes differed for different organic food items. Frequent purchase of organic milk, eggs, or meats tended to be associated with a lower risk of diabetes (Table 4). The multivariable-adjusted ORs (95% CIs) were 0.74 (0.46, 1.22), 0.67 (0.41, 1.08), and 0.66 (0.34, 1.28) (*p* = 0.04 for trend) for participants who purchased organic milk rarely, sometimes, and most of the time/always, respectively, compared with participants who never purchased organic milk. The multivariable-adjusted ORs (95% CIs) were 0.95 (0.63, 1.43), 0.84 (0.48, 1.48), and 0.47 (0.27, 0.83) (*p* = 0.01 for trend) for participants who purchased organic eggs rarely, sometimes, and most of the time/always, respectively, compared with participants who never purchased organic eggs. The multivariable-adjusted ORs (95% CIs) were 0.67 (0.39, 1.16), 0.78 (0.46, 1.32), and 0.67 (0.31, 1.45) (*p* = 0.09 for trend) for participants who purchased organic meats rarely, sometimes, and most of the time/always, respectively, compared with participants who never purchased organic meats. The associations were not appreciably changed after an additional adjustment for BMI. Organic fruits, vegetables or poultry purchases also showed an inverse association with diabetes, although the associations with diabetes were not statistically significant.

An association with the purchase of organic foods differed by age, race/ethnicity education, physical activity levels, and diet quality (Supplementary Table S1). The purchase of organic foods was associated with lower odds of diabetes among participants aged 45–65 and older than 65 years (multivariable-adjusted OR 0.69, 95% CI 0.49, 0.95 and OR 0.74, 95% CI 0.56–0.98), among whites (multivariable-adjusted OR 0.76, 95% CI 0.60–0.96), among people with physical activity levels ≥1200 MET-min/week (multivariable-adjusted OR 0.68, 95% CI 0.49–0.95), and among people with better diet quality (multivariable-adjusted OR 0.67, 95% CI 0.50–0.90), although the interactions were not statistically significant. The magnitude of association was virtually not changed in sensitivity analyses among those with undiagnosed diabetes (Supplementary Table S2), or excluding those who had started insulin within one year of diabetes diagnosis, were currently using insulin, and were diagnosed with diabetes under age 30 (Supplementary Table S3).

## 4. Discussion

With data from a nationally representative population, we found an inverse association between the purchase of organic foods and diabetes in U.S. adults. The association was independent of demographic, socioeconomic, dietary, and lifestyle factors. Moreover, the association showed a dose-response manner, with a more frequent purchase of organic foods being associated with lower odds of diabetes. In terms of individual organic food items, the associations were more pronounced for organic milk, eggs, and meats than for organic fruits or vegetables.

Although organic foods are presumed and perceived widely as healthier alternatives to conventional foods, limited empirical evidence is available to support the claim on the health benefits of organic foods [5,9,10,26,27]. At present, very few existing population studies have collected data on organic foods. To our knowledge, the association between organic foods and diabetes was examined in only one previous study, which showed that regular consumption of organic foods was associated with lower odds of type 2 diabetes in a cross-sectional study of a French population [10]. However, only minimal adjustment for confounders (i.e., age, education, occupation, and income) was performed in that study [10]. Whether the observed association was independent of well-established major risk factors for diabetes, such as race/ethnicity, family history of diabetes, smoking, physical activity, and dietary quality, was not assessed in that study [10]. In the present study, because NHANES collected detailed and comprehensive information on health-related topics, we were able to estimate the association between organic foods and diabetes after controlling for confounding by these factors. Moreover, because our study was based on a nationally representative population, our results can be generalized to a broader population. Finally, our study has expanded the findings of the previous study [10] by examining the associations of different organic food items (i.e., fruits, vegetables, milk and other dairy products, eggs, poultry, and meats), separately, with diabetes. The variations in the observed associations across organic food items with diabetes are important for a better understanding of the potential mechanisms for the inverse association between organic foods and diabetes in the previous [10] and the present study.

Previous studies have shown that people who purchase organic food are more likely to have better dietary and lifestyle habits [11,28]. However, after controlling for smoking, alcohol drinking, physical activity, total energy intake, and overall diet quality, the association remained significant in the present study. Moreover, people who purchase organic food usually have a higher socioeconomic status, e.g., higher education level and family income [29]. However, our results remained significant after adjustment for these variables.

There are several possible explanations for the observed inverse association of organic food purchase with diabetes in our study. For vegetables and fruits, it has been reported that organic products have a 30% lower risk of contamination with any detectable pesticide residue than conventional products [9]. Studies have shown that exposure to various currently used pesticides such as organophosphates and pyrethroids is associated with a higher risk of diabetes [30] through mechanistic pathways involving oxidative damage and inflammatory cytokines, which lead to compensatory responses accompanied with reduced insulin signaling in insulin-sensitive organs [31,32]. However, lower pesticide residues, for which fruits and vegetables, but not animal products, are largely the only sources of exposure, cannot fully explain the association for overall organic product purchase in the present study because the association of organic fruit and vegetable intake with diabetes was not significant.

Organic animal foods or products are known to contain reduced amounts of antibiotic residues compared to conventional animal foods or products [33]. Previous studies have shown that chronic antibiotic exposure is associated with a higher risk of type 2 diabetes [34,35] via possible mechanisms including antibiotic-driven changes in insulin sensitivity, glucose tolerance, lipid deposition, and energy harvesting potential by altering the gut microbiota composition [35]. Furthermore, organic animal products may contain less sex steroids or growth hormones used in farm animals compared to conventional animal products. Lastly, studies suggest that organic foods such as eggs, milk, and meats may contain more beneficial unsaturated fatty acids such as omega-3 fatty acids than conventional products [7,8,9,34]. These fatty acids could have beneficial effects on insulin sensitivity and is likely to reduce the risk of diabetes [36]. However, it is questionable whether a small difference at relatively low intake levels can result in a meaningful difference in disease risk. Further investigation is warranted to determine the role of these chemicals or nutrients in mediating the health effects of organic foods.

The major strength of this population-based study is the use of a nationally representative sample, which facilitates the generalization of the findings to the general population in the United States. In addition, with the detailed data collected in the NHANES, we were able to control potential confounding effects from a variety of demographic, socioeconomic, and lifestyle factors. This study has some limitations. First, the NHANES was a cross-sectional study. As a result, we could not establish a temporal relation and causality for the association between organic foods and diabetes. However, in the situation of reverse causation (i.e., diabetes patients started to use more organic foods), the direction of association would have been towards null or even positive, which could not explain the observed significant and inverse associations in this study. Second, we were unable to distinguish between type 1 diabetes and type 2 diabetes. However, because type 2 diabetes constitutes 90% or more of all diabetes in adults [37], the observed association was likely to be largely reflected by the association of organic foods with type 2 diabetes. Furthermore, we did a sensitivity analysis excluding those who possibly had type 1 diabetes, and the results were not appreciably changed. Third, people with higher socioeconomic status and more organic food use might be more health conscious; thus, early screening and detection of diabetes were possible [38]. However, the OR remained virtually unchanged after we excluded those with already diagnosed diabetes. Fourth, we did not have information on the exact amount of organic food consumption for each participant. Organic food purchase was a proxy of organic food consumption. It is possible that the frequency of organic food purchase does not exactly reflect the frequency of organic food consumption at the individual level. In addition, there has been increasing interest in organic foods over the past decades. Because NHANES data on organic food purchase were collected around 10 years ago, future studies are warranted to determine the health effects of organic food purchase and consumption in the current population. Finally, although we have adjusted for a variety of potential confounders, residual confounding is still possible.

In conclusion, in a nationally representative population, our study has shown that frequent purchase of organic foods, in particular, organic milk, eggs, and meats, was inversely associated with diabetes in adults in the United States. Further investigation is needed to comprehensively evaluate the long-term effects of organic food consumption on chronic diseases, including diabetes.

## Figures and Tables

**Table 1:** Characteristics among 8199 participants in the National Health and Nutrition Examination Survey (NHANES) 2007–2010, according to their purchase of organic foods.

	Purchased any Organic Food in the Past 30 Days		
Characteristics	No	Yes	*p*-Value
No. of participants	5300	2899	
Age, years	47.7 (0.35)	46.4 (0.54)	0.02
Gender			
Male	50.0 (0.78)	40.9 (0.89)	<0.001
Female	50.0 (0.78)	59.1 (0.89)	
Race/ethnicity, %			
Non-Hispanic white	68.6 (2.85)	76.3 (1.82)	<0.001
Non-Hispanic black	12.6 (1.31)	7.4 (0.82)	
Hispanic	14.2 (2.10)	9.8 (1.04)	
Other	4.6 (0.58)	6.5 (0.86)	
Education, %			
Less than high school	22.1 (0.84)	10.1 (1.01)	<0.001
High school	28.2 (1.07)	16.3 (1.04)	
College or above	49.7 (1.38)	73.6 (1.46)	
Ratio of family income to poverty, %			
≤1.30	22.7 (1.17)	12.3 (0.88)	<0.001
1.31–3.50	35.1 (1.18)	29.6 (1.47)	
>3.50	35.3 (1.50)	51.6 (1.54)	
Missing	6.9 (0.65)	6.5 (0.75)	
Family history of diabetes, %			
Yes	38.6 (1.00)	33.7 (1.17)	<0.001
No	61.4 (1.00)	66.3 (1.17)	
Smoking status, %			
Non-smoker	52.4 (1.35)	58.5 (1.50)	<0.001
Current smoker	24.2 (1.01)	14.5 (0.87)	
Former smoker	23.4 (0.78)	27.0 (1.18)	
Alcohol intake *, %			
Non-drinker	76.0 (1.14)	66.3 (1.32)	<0.001
Moderate drinker	8.4 (0.53)	12.5 (0.89)	
Heavy drinker	15.6 (0.88)	21.3 (1.28)	
Physical activity, MET-min/week			
<600	40.0 (0.87)	30.1 (1.43)	<0.001
600–1199	9.4 (0.45)	13.9 (0.87)	
≥1200	50.6 (1.01)	56.0 (1.37)	
Total energy intake (kcal/day)	2187 (24)	2123 (18)	0.03
Healthy Eating Index 2010 score	47.6 (0.30)	54.0 (0.50)	<0.001
BMI categories, %			
Normal/underweight	26.0 (0.87)	37.1 (1.81)	<0.001
Overweight	35.5 (1.01)	31.4 (1.16)	
Obesity	37.8 (0.81)	30.8 (1.31)	
Missing	0.6 (0.1)	0.6 (0.16)	

Values are means (SE) or percentages (SE) and are weighted. Abbreviations: BMI, body mass index; MET, metabolic equivalent. * Non-drinker: 0 g/day; Moderate drinker: 0.1–28 g/day for men and 0.1–14 g/day for women; Heavy drinker: ≥28 g/day for men and ≥14 g/day for women.

**Table 2:** Association between organic food purchase and diabetes among 8199 participants, NHANES 2007–2010.

	Purchased any Organic Food in the Past 30 Days		
	No	Yes	*p*-Value
No. of diabetes cases/participants	875/5300	343/2899	
Model 1 ^†^	1.00 (reference)	0.65 (0.56, 0.75) *	<0.001
Model 2 ^‡^	1.00 (reference)	0.80 (0.68, 0.93)	0.004
Model 3 ^§^	1.00 (reference)	0.80 (0.69, 0.94)	0.01

* Odds ratio (95% confidence intervals). ^†^ Multivariable model 1: adjusted for age (years) and gender. ^‡^ Multivariable model 2: multivariable model 1 plus race/ethnicity, education, ratio of family income to poverty, family history of diabetes, smoking status, alcohol intake, physical activity, total energy intake, and HEI-2010 score. ^§^ Multivariable model 3: multivariable model 2 plus BMI. All of the covariates were either continuous or categorized as in Table 1.

**Table 3:** Association between the frequency of purchase of organic foods and diabetes among 3940 participants, NHANES 2007–2008.

	Frequency of Purchase of Organic Foods				
	Never	Rarely	Sometimes	Most of the Time/Always	*p* for Trend
No. of diabetes cases/participants	455/2612	46/300	84/693	35/335	
Model 1 ^†^	1.00 (reference)	0.81 (0.58, 1.15) *	0.74 (0.51, 1.07)	0.60 (0.29, 1.27)	0.02
Model 2 ^‡^	1.00 (reference)	0.96 (0.71, 1.30)	0.84 (0.59, 1.21)	0.72 (0.35, 1.45)	0.11
Model 3 ^§^	1.00 (reference)	0.90 (0.64, 1.28)	0.82 (0.57, 1.19)	0.76 (0.38, 1.54)	0.12

* Odds ratio (95% confidence intervals). ^†^ Multivariable model 1: adjusted for age (years) and gender. ^‡^ Multivariable model 2: multivariable model 1 plus race/ethnicity, education, ratio of family income to poverty, family history of diabetes, smoking status, alcohol intake, physical activity, total energy intake, and HEI-2010 score. ^§^ Multivariable model 3: multivariable model 2 plus BMI. All of the covariates were either continuous or categorized as in Table 1.

**Table 4:** Association between the frequency of purchase of different organic foods and diabetes among 4250 participants, NHANES 2009–2010.

	Frequency of Purchase of Organic Foods				
	Never	Rarely	Sometimes	Most of the Time/Always	*p* for Trend
**Organic fruits**					
No. of diabetes cases/participants	437/2848	37/385	85/703	37/314	
OR (95% CI) *	1.00 (reference)	0.80 (0.49, 1.30)	0.72 (0.49, 1.05)	0.91 (0.52, 1.61)	0.16
**Organic vegetables**					
No. of diabetes cases/participants	443/2872	36/353	82/701	37/325	
OR (95% CI) *	1.00 (reference)	0.74 (0.46, 1.19)	0.87 (0.63, 1.19)	0.85 (0.49, 1.50)	0.31
**Organic milk and dairy products**					
No. of diabetes cases/participants	498/3301	38/351	37/305	23/292	
OR (95% CI) *	1.00 (reference)	0.74 (0.46, 1.22)	0.67 (0.41, 1.08)	0.66 (0.34, 1.28)	0.04
**Organic eggs**					
No. of diabetes cases/participants	505/3,346	37/293	32/277	22/329	
OR (95% CI) *	1.00 (reference)	0.95 (0.63, 1.43)	0.84 (0.48, 1.48)	0.47 (0.27, 0.83)	0.01
**Organic poultry**					
No. of diabetes cases/participants	504/3328	33/310	38/393	23/219	
OR (95% CI) *	1.00 (reference)	0.87 (0.55, 1.35)	0.71 (0.41, 1.22)	0.80 (0.44, 1.48)	0.20
**Organic meats**					
No. of diabetes cases/participants	510/3395	28/322	42/354	14/172	
OR (95% CI) *	1.00 (reference)	0.67 (0.39, 1.16)	0.78 (0.46, 1.32)	0.67 (0.31, 1.45)	0.09

* Adjusted for age, gender, race/ethnicity, education, ratio of family income to poverty, family history of diabetes, smoking status, alcohol intake, physical activity, total energy intake, HEI-2010 score, the consumption of the corresponding food group, and BMI. All of the covariates were either continuous or categorized as in Table 1.

## Supplementary Materials

The following are available online at http://www.mdpi.com/2072-6643/10/12/1877/s1, Figure S1: Flowchart for participant inclusion and exclusion, Table S1: Stratified analyses for the association between organic food purchase and diabetes among 8199 participants, NHANES 2007–2010, Table S2: Association between organic food purchase and diabetes among 7221 participants (excluding those with already diagnosed diabetes), NHANES 2007–2010, Table S3: Association between organic food purchase and diabetes among 7919 participants excluding those who were suspected for type 1 diabetes (diagnosed with diabetes under age 30, started insulin within one year of diabetes diagnosis, and currently using insulin), NHANES 2007–2010.
