# Supplementary material for: Inverse Association between Organic Food Purchase and Diabetes Mellitus in US Adults
Source: Nutrients. 2018 Dec 3;10(12):1877. doi: 10.3390/nu10121877 (PMC6316834; doi:10.3390/nu10121877)
Supplement: Supplementary file 1 [file nutrients-10-01877-s001.pdf]

Table S1. Stratified analyses for the association between organic food purchase and diabetes among 8199 participants, NHANES 2007-2010 *.

	No. of diabetes cases/participants	Ever purchased any organic food in the past 30 days			
		No	Yes	*p*-value	*p* for interaction
**Age**					
20-45	141/3,392	1.00 (reference)	1.18(0.74, 1.88)	0.48	0.85
45-65	565/2,910	1.00 (reference)	0.69(0.49, 0.95)	0.03	
>65	512/1,897	1.00 (reference)	0.74(0.56, 0.98)	0.04	
**Gender**					
Male	577/3,815	1.00 (reference)	0.81(0.63, 1.06)	0.13	0.82
Female	641/4,384	1.00 (reference)	0.82(0.63, 1.07)	0.14	
**Race/ethnicity**					
White	490/4,114	1.00 (reference)	0.76(0.60, 0.96)	0.02	0.50
Non-white	728/4,085	1.00 (reference)	0.90(0.66, 1.23)	0.50	
**Education**					
Less than high school	456/2,183	1.00 (reference)	0.89(0.61, 1.30)	0.56	0.60
High school	293/1,928	1.00 (reference)	0.93(0.61, 1.41)	0.73	
College or above	469/4,088	1.00 (reference)	0.70(0.49, 1.01)	0.06	
**Ratio of family income to poverty**					
≤1.3	379/2,324	1.00 (reference)	0.92(0.69, 1.22)	0.56	0.85
>1.3-3.5	468/2,825	1.00 (reference)	0.92(0.69, 1.23)	0.57	
>3.5	264/2,368	1.00 (reference)	0.82(0.56, 1.20)	0.31	
**Physical activity, MET-min/week**					
<600	701/3,339	1.00 (reference)	0.82(0.64, 1.06)	0.13	0.58

≥600-1199	114/862	1.00 (reference)	1.23(0.70, 2.16)	0.46	
≥1200	403/3,998	1.00 (reference)	0.68(0.49, 0.95)	0.03	
**HET-2010 score**					
Lower HEI-2010 score	603/4,313	1.00 (reference)	0.94(0.79, 1.12)	0.45	0.93
Higher HEI-2010 score	615/3,886	1.00 (reference)	0.67(0.50, 0.90)	0.01	

*Adjusted for age, gender, race/ethnicity, education, ratio of family income to poverty, family history of diabetes, smoking status, alcohol intake, physical activity, total energy intake, HEI-2010 score and BMI. All of the covariates were either continuous or categorized as in table 1.

Table S2. Association between organic food purchase and diabetes among 7221 participants (excluding those with already diagnosed diabetes), NHANES 2007-2010.

	**Ever purchased any organic food in the past 30 days**		
	**No**	**Yes**	***p*-value**
No. of diabetes cases/participants	177/4,602	63/2,619	
Model 1 †	1.00 (reference)	0.52 (0.36, 0.75) *	0.001
Model 2 ‡	1.00 (reference)	0.67 (0.46, 0.99)	0.04
Model 3 §	1.00 (reference)	0.68 (0.46, 1.01)	0.052

*Odds ratio (95% confidence intervals). † Multivariable model 1: adjusted for age (years) and gender. ‡ Multivariable model 2: multivariable model 1 plus race/ethnicity, education, ratio of family income to poverty, family history of diabetes, smoking status, alcohol intake, physical activity, total energy intake, and HEI-2010 score. § Multivariable model 3: multivariable model 2 plus BMI. All of the covariates were either continuous or categorized as in table 1.

Table S3. Association between organic food purchase and diabetes among 7919 participants excluding those who were suspected type 1 diabetes (diagnosed with diabetes under age 30, started insulin within 1 year of diabetes diagnosis, and were currently using insulin), NHANES 2007-2010.

	**Ever purchased any organic food in the past 30 days**		
	**No**	**Yes**	***p*-value**
No. of diabetes cases/participants	680/5,105	260/2,814	
Model 1 †	1.00 (reference)	0.62 (0.52, 0.74) *	<0.001
Model 2 ‡	1.00 (reference)	0.79 (0.66, 0.94)	0.01
Model 3 §	1.00 (reference)	0.80 (0.66, 0.96)	0.02

* Odds ratio (95% confidence intervals). † Multivariable model 1: adjusted for age (years) and gender. ‡ Multivariable model 2: multivariable model 1 plus race/ethnicity, education, ratio of family income to poverty, family history of diabetes, smoking status, alcohol intake, physical activity, total energy intake, and

HEI-2010 score. § Multivariable model 3: multivariable model 2 plus BMI. All of the covariates were either continuous or categorized as in table 1.

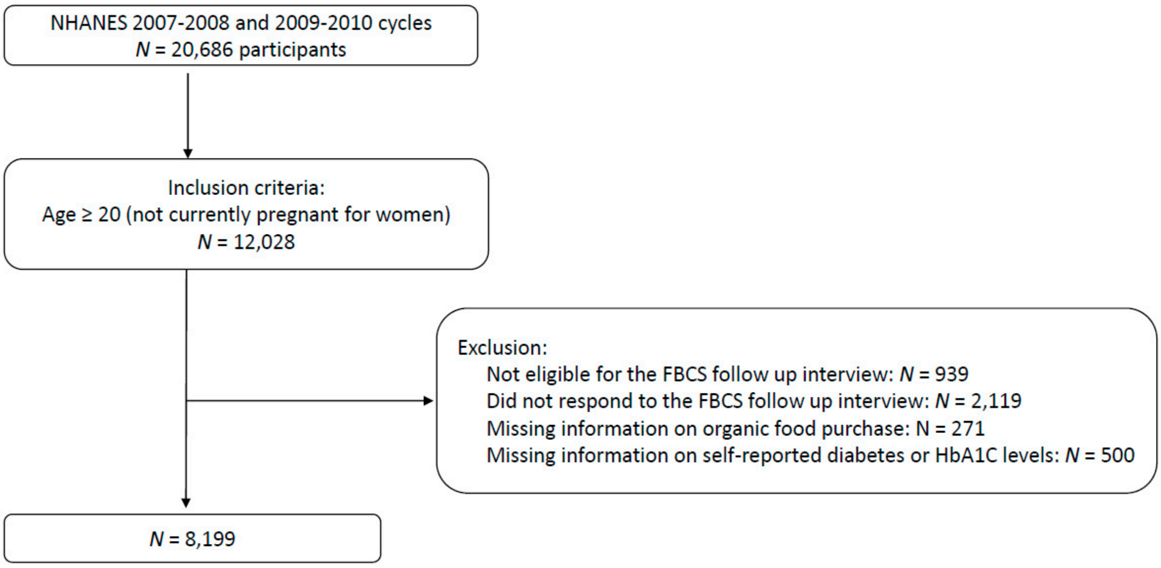

Figure S1. Flow chart for participant inclusion and exclusion.
